# Respiratory Syncytial Virus Elicits Glycolytic Metabolism in Pediatric Upper and Lower Airways

**DOI:** 10.3390/v17050703

**Published:** 2025-05-14

**Authors:** Armando S. Flores-Torres, Svetlana Rezinciuc, Lavanya Bezavada, Barry L. Shulkin, Stephania A. Cormier, Heather S. Smallwood

**Affiliations:** 1 Department of Pediatrics, University of Tennessee Health Science Center, Memphis, TN 38103, USA; aflorest@stjude.org (A.S.F.-T.); rezinciuc.sveta@gmail.com (S.R.); lavanya.bezavada@stjude.org (L.B.); 2 Department of Biological Sciences, Louisiana State University, Pennington Biomedical Research Center, Baton Rouge, LA 70803, USA; barry.shulkin@stjude.org; 3 Department of Diagnostic Imaging, St. Jude Children’s Research Hospital, Memphis, TN 38105, USA; stephaniacormier@lsu.edu

**Keywords:** RSV, respiratory syncytial virus, metabolism, airway epithelial cells, pediatric, glycolysis, oxidative phosphorylation, viral infection, bioenergetics, infant

## Abstract

Respiratory syncytial virus (RSV) is the leading cause of lower respiratory tract viral infection in infants and causes around 60,000 in-hospital deaths annually. Emerging evidence suggests that RSV induces metabolic changes in host cells to support viral replication, presenting a potential target for therapeutic intervention. To investigate RSV-driven metabolic changes in situ, we combined positron emission tomography (PET), live-cell bioenergetics, and metabolomic profiling in the upper and lower airways of children. PET imaging revealed persistent, hyper-glycolytic regions in the lungs of RSV-infected children. Bioenergetic analysis of freshly collected nasopharyngeal aspirates from infants showed live upper respiratory cells (URCs) infected with RSV in situ exhibited significantly higher levels of glycolysis, glycolytic capacity, glycolytic reserves, and mitochondrial respiration than uninfected controls. Metabolomic analysis of nasopharyngeal fluids from these patients revealed distinct metabolic signatures, including increased citrate and malate, and decreases in taurine. In vitro infection of pediatric nasopharynx tissue-derived multicellular epithelial cultures (TEpiCs) and bronchial epithelial cells further confirmed RSV-induced increases in glycolysis. Together, these findings demonstrate that RSV infection induces hypermetabolism in both upper and lower primary airways in situ, supporting the potential of host-targeted metabolic interventions as a therapeutic strategy—particularly in vulnerable populations such as infants for whom vaccines are not currently available.

## 1. Introduction

Respiratory syncytial virus (RSV) is a non-segmented, negative-sense, single-stranded RNA virus, classified within the *Paramyxoviridae* family that infects predominantly infants and most children by the age of 2 years and reemerges as a serious lower respiratory tract illness in the elderly [1]. The etiology and exacerbation of RSV have been attributed to host genetic factors [2,3], innate and adaptive immune responses [4,5,6,7], pathophysiological factors [8], and an immature immune system with delayed adaptive responses [9]. Beyond the immediate impact, RSV infection during the initial three years of life is linked to long-lasting respiratory complications such as asthma development, recurrent wheezing, diminished lung function, and allergic sensitization [10]. Furthermore, RSV serious lower respiratory tract illness is associated with asthma and chronic obstructive pulmonary disease exacerbations [11,12]. Two RSV vaccines were recently approved for older adults and pregnant women, but no vaccines currently exist for children [13].

Viruses, including those that target the respiratory tract such as the influenza virus, and Severe Acute Respiratory Syndrome Coronavirus 2 (SARS-CoV-2), induce metabolic reprogramming to ensure an ample supply of energy and biomolecules for their replication and survival, and to manipulate the host immune response [14,15]. Initial mass spectrometry studies of urine, sera, and nasopharyngeal aspirates indicate RSV alters metabolite profiles [16,17,18,19]. In vitro RSV infection studies in A549 cells showed upregulation of glycolytic and tricarboxylic acid cycle (TCA) metabolites [20,21], and A549, HEp2, and HeLa responded by moving from oxidative phosphorylation (OXPHOS) to glycolysis [22,23]. However, cell lines are subject to the Warburg effect metabolic phenotype characterized by the preferential conversion of glucose to lactate, even in the presence of oxygen, making it difficult to interpret viral-induced metabolic reprogramming [24,25,26,27]. One study used human small alveolar epithelial cells, and while it is unclear how many donors were used, they found RSV infection increased glycolytic activity, which was mediated by HIF-1α [26]. More recently, glucose flux through glycolysis was significantly increased in cultured epithelial cells from nasal brushings of male infants who were naturally infected with RSV [27]. These studies highlight the need to determine whether RSV alters metabolism in situ and to develop pediatric airway models from primary cells to better understand the mechanisms underlying virus-induced changes and develop treatments to target them.

In a retrospective clinical study, we found RSV-induced hyper glycolytic lesions in the lungs of infected pediatric patients [28]. We then aimed to determine whether RSV reprogrammed metabolism in the upper respiratory tract and to identify what specific pathways were impacted. To achieve this, we employed bioenergetics to provide a window into the critical functions driving cell signaling, activation, biosynthesis, and viral replication. This appears to be the first quantification of live cell metabolism of nasopharyngeal aspirates (NPAs) immediately after collection. RSV infection significantly increased glycolysis and mitochondrial respiration in these upper respiratory cells (URCs) compared to uninfected infants. Metabolomics of matched upper respiratory fluids from these patients identified six metabolites significantly altered by RSV. To determine whether this was entirely mediated by immune cells, we shifted to a new in vitro infection model. We quantified bioenergetics of RSV-infected pediatric nasopharynx tissue-derived multicellular epithelial cultures (TEpiCs) compared to traditional human bronchial epithelial cells (HBECs) obtained from a pediatric donor. We found glycolysis was significantly increased in both TEpiCs and HBECs. Collectively, these data indicate RSV reprograms host metabolism in situ and specifically reprograms epithelial cells, this information advances our understanding of RSV-host interactions and has implications for the development of metabolic-targeting treatments [28]. To our knowledge this is the first quantification of the bioenergetics of live NPA cells from patients, use of nasopharyngeal tonsil tissue epithelium cultures as an in vitro model of RSV infection, and evaluation of RSV-induced metabolic changes in three different relevant primary airway cell types (URCs, TEpiCs, and HBECs).

## 2. Materials and Methods

### 2.1. Subjects and Study Procedures

Inclusion criteria required participants who met the clinical case definition of RSV infection or were asymptomatic. This study was conducted in compliance with 45 CFR46 and the Declaration of Helsinki. Institutional Review Boards of the University of Tennessee Health Science Center/Le Bonheur Children’s Hospital approved the study. Participants provided nasal swabs and nasal lavages after enrollment. Each subject was assigned a unique study identification number (SID) to track samples and diagnostic results. St. Jude Children’s Research Hospital approved the retrospective study of respiratory infected patients who received PET scans.

### 2.2. Infected Patient PET Scans

These previously published data and methods were reanalyzed with respect to RSV [28]. Briefly, patients with normal glucose levels received I.V. injections of fluorodeoxyglucose (FDG) after fasting. Relaxed, prone patients remained in a quiet, dark room. One hour later, transmission computed tomography (CT) and positron emission tomography (PET) images were captured with a GE Discovery LS PET/CT system or 690 PET/CT system (GE Medical Systems, Waukesha, WI, USA). Vendor-supplied software was used for reconstruction, and standardized uptake values were determined.

### 2.3. Pediatric Nasal Pharyngeal Aspirates

Uninfected controls (*n* = 2) and RSV-infected subjects (*n* = 4) were included in the study, with RSV-positive cases diagnosed as either RSV-A or RSV-B. Subjects ranged from 8 to 67 weeks old. Symptom duration varied from 3 to 21 days. After enrollment, subjects were swabbed and nasal rinses obtained. Clinical diagnostics were performed including antigen (Ag) test and reverse transcription quantitative polymerase chain reaction (RT-qPCR). Nasal aspirates were obtained at enrollment, placed on ice immediately and small aliquot removed for diagnostics. The aspirates were immediately transported on ice to the research laboratory and cells separated by gently centrifuging. The supernatant was stored at −80 °C, and the viability and number of URCs were determined using acridine orange and propidium iodide. Across all samples, URC viability ranged from 67% to 84%, with cell concentrations ranging from 1.59 × 10^6^ to 1.45 × 10^7^ cells/mL.

### 2.4. Cells and Virus

HBECs from a healthy pediatric donor were obtained from Lonza (Walkersville, MD, USA). Cells were expanded and cultured in Bronchial Epithelial Growth Medium (BEGM™), consisting of BEBM™ supplemented with a SingleQuots™ kit (Lonza, Walkersville, MD, USA). RSV strain A2 was generously provided by Dr. Asunción Mejías from St Jude Children’s Research Hospital.

### 2.5. Pediatric Nasopharynx Tissue-Derived Multicellular Epithelium Cultures (TEpiCs)

Pediatric patients, ranging from 4 to 8 years old, donated their excised tonsils, which were removed as part of standard care procedures for conditions such as tonsillitis and sleep apnea. Excised tissues were placed on ice and processed within 1 h. Multicellular epithelium 2D cultures generally followed a protocol developed as a human model for influenza infection and generously provided by Dr. Richard Webby [29]. Briefly, processing followed standard procedures including decontamination with antimycotic/antibiotics, dissection, mechanical disruption, enzymatic digestion with collagenase II for 2 h at 37 °C, and cell straining to get a single cell suspension. The cells were then enumerated and cryopreserved. Thawed cells were cultured in BEGM until they reached 80% of confluency. Validation of epithelium cultures was confirmed by flow cytometry using EpCAM and CD45 markers on the Aurora spectral flow cytometer, and the data were analyzed with FlowJo (Tree Star, San Carlos, CA, USA).

### 2.6. Cell Bioenergetics

URCs (200,000 per well) were immediately seeded in XFe96 plates following the manufacturer’s cell suspension protocol (Agilent, Santa Clara, CA, USA). The glycolytic stress test and mitochondrial stress test were performed in separate wells in the same plate to expedite measurements. One to six wells of technical replicates were run per patient sample.

For HBECs and TEpiCs, XFe96 plates were coated with 50 µg/mL rat collagen prior to seeding the cells (20,000 cells per well). The cells were then infected with RSV-A2 at an MOI of 3 and 10, respectively, and incubated for 2 h. After the infection, the unbound virus was washed away, and the cells were incubated in BEGM for an additional 24 h. Following this incubation, HBECs were subjected to glycolysis stress test, while TEpiCs were subjected to both glycolysis stress test and mitochondrial stress test according to the manufacturer’s instructions. Five to seven wells of technical replicates were run per experimental treatment.

The Seahorse Glycolytic Stress Test assesses cellular glycolysis by sequentially adding glucose (10 mM), oligomycin (2 µM), and 2-deoxy-D-glucose (50 mM) while measuring the extracellular acidification rate (ECAR). Basal glycolysis is determined after glucose addition, glycolytic capacity is measured following oligomycin treatment (which inhibits ATP synthase and forces cells to rely on glycolysis), and glycolytic reserve is calculated as the difference between glycolytic capacity and basal glycolysis. The Seahorse Mitochondrial Stress Test evaluates oxidative phosphorylation by measuring the oxygen consumption rate (OCR) in response to sequential injections of oligomycin (2 µM) (ATP synthase inhibitor), FCCP (1 µM) (an uncoupler that drives maximal respiration), and rotenone/antimycin A (0.5 µM each) (complex I and III inhibitors, shutting down mitochondrial respiration). This assay quantifies key parameters such as basal respiration, ATP-linked respiration, maximal respiratory capacity, and spare respiratory capacity.

Prior to experimentation, we optimized cell seeding density and performed inhibitor titrations following the manufacturer’s best practices guidelines to ensure optimal signal detection and assay performance. Cells were washed with Seahorse XF Assay Medium and incubated in a 37 °C non-CO_2_ incubator for 45 min to 1 h before measurement. A sensor cartridge was hydrated in calibrant for 12–24 h in a non-CO_2_ incubator at 37 °C before experiments. DNA per well was quantified with CyQUANT (Thermo Scientific, Waltham, MA, USA) and used for data normalization.

### 2.7. Metabolomics of Nasopharyngeal Fluids

Metabolites were extracted from nasopharyngeal fluids and subjected to ultra-performance liquid chromatography–high-resolution mass spectrometry (UPLC–HRMS) analysis. Briefly, metabolites were solvent-extracted, solvent-evaporated, resuspended in water, and placed in a chilled autosampler for mass spectrometric analysis. Aliquots (10 µL) were injected through a Synergi 2.5-micron reverse-phase Hydro-RP 100, 100 × 2.00 mm LC column (Phenomenex, Torrance, CA, USA) and introduced into the MS via an electrospray ionization source conjoined to an Exactive™ Plus Orbitrap Mass Spectrometer (Thermo Scientific). We used full-scan mode with negative ionization mode (85–1000 *m*/*z*), 3 kV spray voltage, 10 psi flow rate at 320 °C, 3e6acquisition gain control, 140,000 resolutions with scan windows of 0 to 9 min at 85 to 800 m/z and 9 to 25 min at 110 to 1000 *m*/*z* and solvent gradient [30]. Data files generated by Xcalibur (Thermo Scientific) were converted to open-source mzML format using ProteoWizard [31,32]. Maven (mzRoll) software (version 3.9.9, Apache Software Foundation, Wakefield, MA, USA) automatically corrected total ion chromatograms based on the retention times for each sample and selected unknown peaks [33,34]. Metabolites were manually identified and integrated using known masses (±5 ppm mass tolerance) and retention times (Δ ≤ 1.5 min).

### 2.8. Statistical Analysis

**Figure 1:**
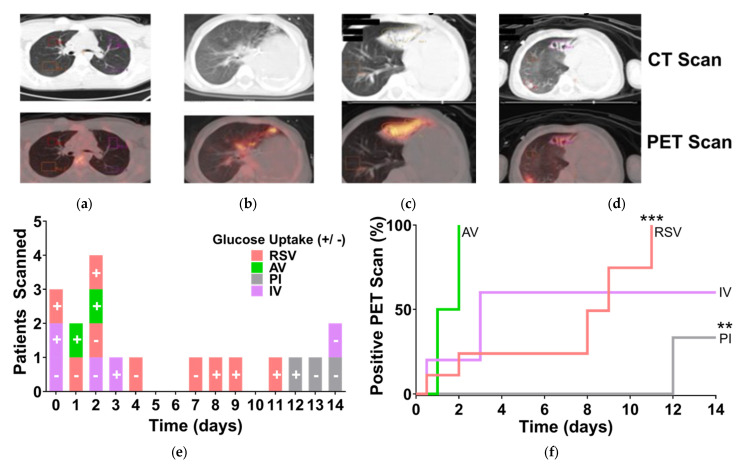
Children with RSV Display Hypermetabolic Activity in their Lungs for Extended Periods. We performed a retrospective study of pediatric patients who received PET-CT scans within 15 days of clinical diagnosis with respiratory viral infection. Acceptance criteria included confirmation of clinical diagnosis with RT-qPCR within 15 days of the PET scan. Whole-body transmission CT and PET images were obtained after patients with normal blood glucose fasted for 4 or more hours. FDG was administrated intravenously (5.5 MBq/kg), followed by a 1 h uptake period. (**a**–**d**) Representative images with CT scans (top) and PET scans (bottom). (**a**) Uninfected patient: PET-CT scan showing no hypermetabolic activity in the lungs. (**b**) RSV-positive patient: PET-CT scan showing increased FDG uptake indicative of hypermetabolic activity in the lungs. (**c**) RSV-positive patients on day 7 after diagnosis shows continued increased FDG uptake, indicating persistent hypermetabolic activity. (**d**) The same patent’s subsequent PET-CT scan on 198 days after diagnosis showing resolved FDG uptake. (**e**) The number of patients scanned is plotted against time in days from RT-qPCR to PET scan, with “0” indicating the scan and RT-qPCR were performed on the same day. Subjects are colored by viral group and indicated with “+” for positive and “−” for negative glucose uptake in the PET scan. (**f**) Kaplan–Meier product limit method was used to create curves for the infected subjects at risk for hypermetabolism diagnosed by PET scan, and the curves were compared with log-rank tests. Pearson’s correlation test was performed on each event risk curve. Both PI- and RSV-infected groups had significant temporal correlations (associated *p*-µs of 0.0078 and 0.0250, respectively) represented by asterisks (** *p* < 0.01, *** *p* < 0.001), with Pearson’s r values of −0.9922 and −0.8165 and r2 = 0.9845 and 0.6667, respectively. AV: Adenovirus; CT: Computed Tomography; FDG: Fluorodeoxyglucose; IV: Influenza Virus; PET: Positron Emission Tomography; PI: Parainfluenza virus; RSV: Respiratory Syncytial Virus.

For PET imaging analysis (Figure 1), Pearson’s correlation coefficients (r) and coefficient of determination (R^2^) were calculated to assess relationships between glucose uptake and time since diagnosis. Kaplan–Meier survival curves were generated using the product limit estimator to estimate the probability of persistent FDG uptake over time. Log-rank (Mantel–Cox) tests were used to compare survival curves between viral infection groups. Significance thresholds are indicated in the figure legends.

**Figure 2:**
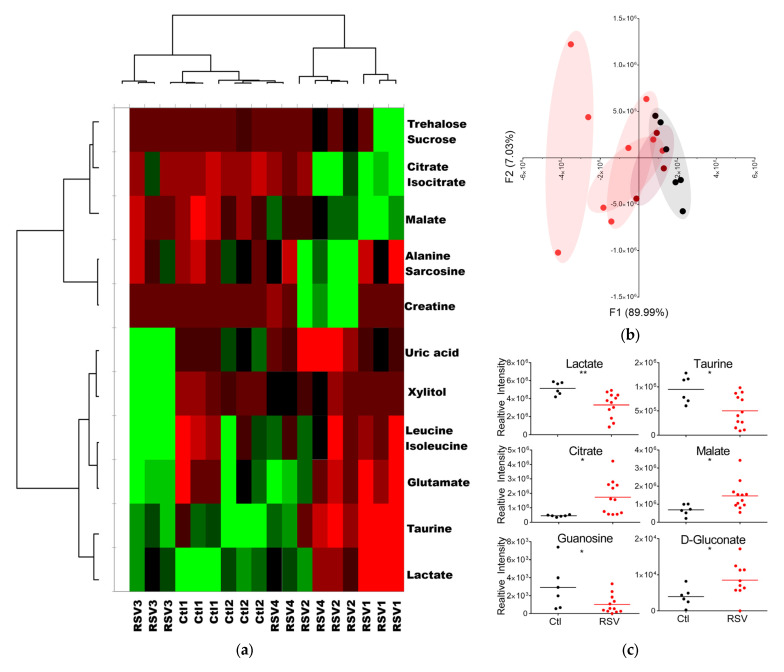
RSV infection changes metabolite composition in upper respiratory fluids. (**a**) Metabolites were extracted from 50 µL NPA supernatant and subjected to UPLC-HRMS metabolomics analysis three times per sample. Metabolites were manually identified and integrated using known masses (±5 ppm mass tolerance) and retention times (Δ ≤ 1.5 min). Peak intensity was normalized to the sum of peak intensity. Metabolites were K-means clustered followed by ascendant hierarchical clustering based on Euclidian distances and nondynamic metabolites excluded (0.25 < std dev). Metabolite clusters were also represented via dendrograms displayed vertically for metabolites and horizontally for patients. The data values of the permuted matrix were replaced by corresponding color intensities based on interquartile range with color scale of red to green through black, resulting in a heat map. (**b**) Principal Component Analysis (PCA) of metabolite profiles showing clustering of RSV-infected patients (red), uninfected controls (black), and RSV patient 3 (maroon) along components F1 and F2, which account for 89.99% and 7.03% of the total variance, respectively. (**c**) Relative intensity data for each metabolite was graphed in Prism. Graphs represent the mean and standard deviation of each patient group with asterisks symbolizing *p*-values determined using two-tailed unpaired *t*-tests. *p*-values: 0.05 (*) and <0.01 (**).

Multivariate statistical analysis for MS/MS data (Figure 2) was performed using XLSTAT OMICS (Addinsoft, New York, NY, USA) with Excel (Microsoft Corporation, Redmond, WA, USA). To ensure observations were directly comparable and to account for respiratory secretion concentrations, peak intensity was normalized to total intensities. These data were independently k-means clustered followed by ascendant hierarchical clustering based on Euclidian distances. Data values of the permuted matrix were replaced by corresponding color intensities based on interquartile range with a color scale of red to green through black. Unsupervised multivariate principal component analysis (PCA) was performed, and the difference in metabolite concentrations per group was determined using one-way ANOVA with Benjamini–Hochberg post hoc correction. Significant differences were detected using Tukey’s honest significant difference (HSD) test for multiple comparisons. Mean intensity data and standard deviation for each metabolite were graphed in Prism (GraphPad, San Diego, CA, USA) and tested for significance using unpaired *t*-test.

**Figure 3:**
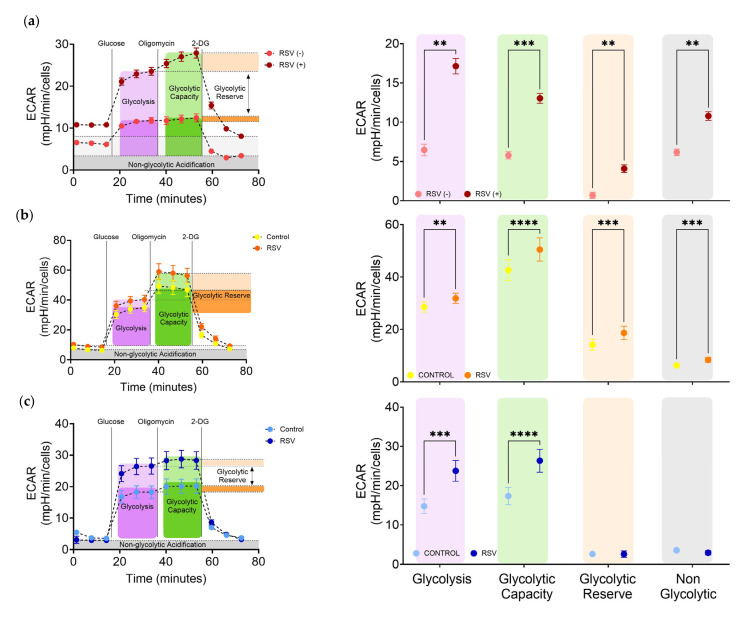
RSV-induced Glycolytic Reprogramming in Upper and Lower Airways Cells. Quantification of glycolytic parameters in control and RSV-infected patients or RSV-infected cells was performed using the Seahorse XF96 Extracellular Flux Analyzer. ECAR was measured in real-time upon sequential additions of glucose (10 mM), oligomycin (2 µM), and 2-deoxyglucose (2-DG; 50 mM). Graphs on the left represent the kinetics after each drug injection, which depict the mean ± SEM of the ECAR (mpH/min/cells). Each column on the right corresponds to a different glycolytic parameter: glycolysis rate (purple), glycolytic capacity (green), glycolytic reserve (orange), non-glycolytic acidification (gray). (**a**) URCs obtained from controls or RSV positive subjects. Results represent the mean ± SD for *n* = 2 biological replicates for negative controls and *n* = 4 biological replicates for RSV-positive subjects. (**b**) TEpiCs were used as uninfected controls or infected with RSV (MOI of 10) for 24 h. Results represent the mean ± SD for *n* = 3 biological replicates. (**c**) HBECs were used as uninfected controls or infected with RSV (MOI of 3) for 24 h. Results represent the mean ± SD for *n* = 3 independent experiments. Statistical significance was determined using a mixed-effects model with repeated measures (REML), followed by Sidak correction for multiple comparisons. The following *p*-values are denoted: ** *p* < 0.01, *** *p* < 0.001, **** *p* < 0.0001. ECAR: Extracellular Cellular Acidification Rate; HBECs: Human Bronchial Epithelial Cells; RSV: Respiratory Syncytial Virus; TEpiCs: Nasopharynx-tissue-derived multicellular epithelial cultures; URCs: Upper Respiratory Cells.

**Figure 4:**
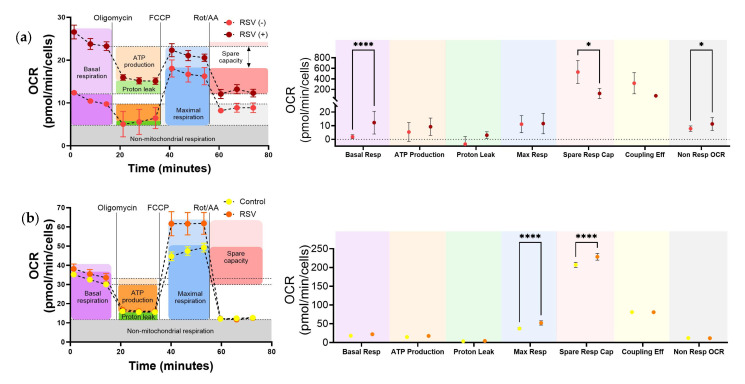
RSV-induced Mitochondrial Reprogramming in Upper Airways Cells and epithelium cultures. Quantification of mitochondrial respiratory parameters in control and RSV-infected patients (left) and RSV-infected TEpiCs (right) was performed using the Seahorse XF96 Extracellular Flux Analyzer. Oxygen consumption rate (OCR) was measured in real-time upon sequential additions of oligomycin (2 µM), FCCP (1 µM), and a mix of rotenone and antimycin A (0.5 µM). Graphs in (**a**) represent the kinetics after each drug injection, which depict the mean ± SEM of the OCR (pmol/min/cells). Agilent Seahorse Wave Desktop software (version 2.6.4.24) was used to calculate each individual component of mitochondrial function and normalize to cell number (**b**). URCs obtained from fresh nasopharyngeal aspirates from RSV negative controls (*n* = 2) and RSV-positive subjects (*n* = 4), depicted on the left. TEpiCs generated from 3 donors were treated with vehicle (control) or infected with RSV at MOI of 10 for 24 h (RSV), depicted on the right. Statistical significance was determined using a mixed-effects model with repeated measures (REML), followed by Sidak correction for multiple comparisons. The following *p*-values are denoted: * *p* < 0.05, **** *p* < 0.0001. RSV: Respiratory Syncytial Virus; OCR: Oxygen Consumption Rate; TEpiCs: Nasopharynx-tissue-derived multicellular epithelial cultures; URCs: Upper Respiratory Cells.

For statistical analysis of cell bioenergetics (Figure 3 and Figure 4), data analysis was performed using Agilent Seahorse Wave software v2.6.1 (Agilent, Santa Clara, CA, USA). A mixed-effects model with repeated measures was used, employing restricted maximum likelihood (REML) estimation. This approach accounts for the correlation between paired samples (e.g., infected and uninfected conditions from the same donor) and inter-donor variability. Post hoc pairwise comparisons were performed using Sidak’s correction for multiple testing. Results are presented as mean ± SD, and significance thresholds are indicated in the figure legends.

## 3. Results

### 3.1. RSV-Induced Hypermetabolism in the Lungs of Pediatric Patients

We previously observed hypermetabolic regions in the tumor free lungs of nine children with respiratory viral infections and found a significant negative monotonic relationship between glucose uptake and time from infection diagnosis in the entire cohort [28]. We performed a new retrospective study of pediatric patients diagnosed with respiratory viral infections confirmed by RT-qPCR who underwent fludeoxyglucose (FDG)-18 positron emission tomography/computed tomography (PET/CT) scans. FDG uptake in tissue is proportional to the metabolic rate of a region, and hypermetabolic lesions, regions, and foci are readily detected with FDG-PET/CT [35]. Representative images of uninfected, RSV-infected, and a subject we followed for 6 months as the RSV-induced hypermetabolic regions subsided can be seen in Figure 1a, b, c, and d, respectively. In this study, we grouped all respiratory viruses together including RSV, IV, adenovirus (AV), or parainfluenza (PI) and then compared the temporal distribution of subjects separated by virus. Patients with glucose uptake were unevenly distributed among respiratory pathogens. This revealed that patients infected with RSV were positive for glucose uptake in the lungs for much longer (Figure 1e).

We performed time-to-event analysis on these groups to determine the proportion of patients who likely had hypermetabolic regions due to respiratory viral infections. We plotted the number of subjects at risk over time from RT-qPCR to PET scan using the product limit estimator method (Kaplan and Meier) to estimate the proportion of infected individuals who likely had glucose uptake in their lungs within 2 weeks of diagnosis. AV-infected patients had a median of 1.5 days, and IV-infected patients had 3 days. By contrast, RSV had a median event time of 9 days. These curves were significantly different by log-rank using the Mantel-Cox test (Figure 1f). We used Pearson’s correlation test to determine the relationship between glucose uptake risk (percent) and time elapsed between diagnosis by RT-qPCR and PET scan. RSV had a significant positive relationship (r value: 0.9117, R2: 0.8312, *p*-value: 0.0006), indicating hypermetabolism in RSV-infected patients’ lungs continued throughout the study interval, with higher associated risk one week after diagnosis.

### 3.2. RSV Infection Alters Metabolite Levels and Profiles in Upper Airway Fluids

To determine whether RSV globally altered metabolism in the upper airways, we performed metabolomics on fluids recovered from nasopharyngeal aspirates collected as previously described [36,37]. This procedure is non-invasive and often performed on infants and children with RSV to clear the sinuses and provide temporary relief [38]. Twelve babies less than 1.3 years of age, five female and seven male, neither intubated nor admitted to the ICU, were enrolled, and medical record data, swabs, and NPA collected. NPA were immediately processed and cell number and viability determined. Five patients were excluded from our analysis due to low cell viability and URC numbers in their NPAs. NPA 240 specimens were immediately separated by centrifugation into nasopharyngeal fluids and URC. Metabolites were extracted from nasopharyngeal fluids and subjected to ultra-performance liquid chromatography–high-resolution mass spectrometry (UPLC–HRMS) analysis using a targeted discovery metabolomics (TDM) approach. We previously determined the masses (±5 ppm mass tolerance) and retention times (Δ ≤ 1.5 min) of 300 metabolites associated with disease pathologies. Using our previously determined masses and retention times, we manually identified and integrated NPA metabolites for targeted discovery and found 35 metabolites in the NPA. To determine the dataset structure and relationships between groups, we used unsupervised multivariate statistical analysis. Metabolites and individuals were clustered independently using k-means clustering followed by ascendant hierarchical clustering based on Euclidian distances. We arranged data matrices according to the clustering with spatial relationships proportional to similarity among patients or metabolites (Figure 2a).

The top horizontal dendrogram separates patients while the vertical groups metabolites, each by how strongly their concentrations correlate. With metabolite peak intensities rearranged according to metabolite clustering, the metabolites are divided into two distinct groups on the *y*-axis (Figure 2a-left dendrogram). These groups show metabolite concentrations depending on RSV infection. The patient samples are also divided into two groups (Figure 2a-top dendrogram). Interestingly, RSV-infected patient 3 (RSV3) grouped with the negative control (Ctl), whereas RSV patients 1, 2, and 4 clustered together (Figure 2a). When we decoded the samples, patient 3 had been symptomatic for 21 days, while the other RSV-infected patients were symptomatic for a week or less. This finding might reflect the kinetics of returning to metabolic homeostasis in the upper airway. However, more data are needed to confirm this idea. The controls and RSV3 group display an inverse pattern of metabolites with a relatively low concentration in the top metabolite cluster and relatively high concentrations in the bottom cluster (Figure 2a). In contrast, RSV patients 1, 2, and 4 exhibit lower concentrations for most metabolites.

To evaluate group trends and sample uniformity as well as identify potential outliers, we used PCA. Variation was explained by F1 and F2 very well, with a cumulative percent variability of 95.57% (Figure 2b). Furthermore, the F1 or F2 squared cosines of each patient observation exceeded 0.5. Again, RSV patient 3 had a closer relationship to controls than patients with more recent symptom onset (Figure 2b maroon). The overlap of RSV patient 3 and controls likely reflects the trajectory of this patient’s recovery. To identify specific metabolic products that change during RSV infection, we compared uninfected controls to RSV-infected patients who were symptomatic for less than a week (acute RSV). Six upper respiratory fluid metabolites were significantly different in patients with acute RSV (Figure 2c). Significant depleted compounds in RSV-infected samples included lactate, taurine, and guanosine. On the other hand, RSV infection triggered increased levels of some metabolites including citrate, D-gluconate and malate compared to non-infected samples.

### 3.3. Increased Glycolysis in Upper Airways Cells Infected by RSV In Situ

To determine the molecular mechanisms driving the global metabolic changes in the airways of infants with community-acquired RSV infections, we evaluated the glycolytic function of live cells from freshly collected nasopharyngeal aspirates. Bioenergetic measurements were performed on Xfe96 bioanalyzer. To determine whether RSV reprogrammed metabolism in situ cells from NPA were immediately plated and the glycolysis stress test performed, which measures the extracellular acidification rate (ECAR) from lactate to quantify glycolytic flux. RSV infection in situ, significantly increased glycolysis (Figure 3a purple). Oxidative phosphorylation was inhibited with oligomycin to force URCs to use glycolysis. RSV infection significantly and dramatically increased the glycolytic capacity or maximum glycolytic rate of URCs (Figure 3a green). Remarkably, URCs from infected patients remained capable of responding to increased energy demands and had significantly higher glycolytic reserve than those from uninfected patients (Figure 3a orange). Infection also significantly increased non-glycolytic acidification, which can come from carbon dioxide in the TCA cycle and is often very high in activated tissue resident macrophages and T cells (Figure 3a gray). These data suggest that community-acquired RSV reprograms upper respiratory cells in situ to dramatically increase glycolysis in the upper airways.

### 3.4. In Vitro RSV Infection Significantly Increased Glycolysis in Upper Airway Epithelium Cultures and Lower Airway Bronchial Epithelial Cells

Fresh nasopharyngeal aspirates contain a mixture of epithelial and immune cells. To evaluate whether RSV reprograms the epithelium of the upper airways we obtained nasopharynx tissue from three pediatric patients. We generated pediatric nasopharynx tissue-derived multi-cellular epithelial cultures (TEpiCs) and used traditional bronchial epithelial cells (HBECs) from a healthy pediatric subject. TEpiCs were evaluated by flow cytometry to confirm they were epithelial cultures (Supplementary Figure S1). Similar to the URCs, RSV infection of TEpiCs significantly increased glycolysis, glycolytic capacity, glycolytic reserve, and non-glycolytic acidification (Figure 3b). In our hands, commercially available HBECs have highly variable bioenergetics after differentiation. Nonetheless, HBECs significantly increased glycolysis and glycolytic capacity after RSV infection (Figure 3c). These results clearly demonstrate that RSV rapidly reprograms 2D multicellular upper airway epithelium cultures inducing a hyper glycolytic state likely reflecting the increased energy demands and biosynthetic needs imposed by infection and shows this model yields similar responses to URCs. A summary of the statistical differences across all metabolic parameters assessed in URCs, TEpiCs, and HBECs is presented in Supplementary Figure S2.

### 3.5. RSV Increases Mitochondrial Respiration in Upper Respiratory Cells Infected In Situ

We then performed a mitochondrial stress test to assess the flux of pyruvate into the TCA cycle and its contribution to mitochondrial respiration by measuring the oxygen consumption rate of URCs (Figure 4a left); these data were subsequently used to calculate key parameters of mitochondrial function (Figure 4a right). First, we measured the basal respiration, which refers to the oxygen consumption necessary to fulfill cellular ATP requirements and displays the cellular energy needs under basal conditions. We found that URCs from RSV-infected patients dramatically increased basal respiration compared to controls (Figure 4a purple). However, the RSV-induced increase in basal respiration did not lead to an increase in ATP production (Figure 4a orange). Additionally, the spare capacity of URCs was decreased in infected infants compared to controls (Figure 4a red). Non-mitochondrial OCR was also significantly increased (Figure 4a, gray). Epithelial cells were detected in fresh nasopharyngeal aspirates, but they only make up about 13% of URCs. To assess the impact of RSV infection on the respiratory epithelium, we evaluated the mitochondrial function of TEpiCs (Figure 4b). Unlike URCs, RSV-infected TEpiCs showed no change in basal respiration but exhibited a significant increase in maximal respiration and spare respiratory capacity (Figure 4b blue and red), suggesting that RSV enhanced the respiratory potential of these cells. Altogether, these data indicate that RSV infection in situ increases respiration at or near the URC maximum respiratory potential without improving mitochondrial ATP production.

## 4. Discussion

RSV is the primary cause of lower respiratory tract viral infection in infants, and it is linked to approximately 60,000 annual in-hospital deaths annually, predominantly in infants [36]. The absence of effective vaccines and treatments for this vulnerable population underscores the urgent need to elucidate the mechanisms driving RSV infection and its severity in infants and children [37]. Viruses frequently exploit host cell metabolism, downregulating oxidative phosphorylation while upregulating glycolysis and glutaminolysis to meet the energetic and biosynthetic demands of virion production [14,38]. Recent research has shown that antiviral drugs targeting host cell metabolism can be effective [28,39,40,41,42]. While metabolic reprogramming during RSV infection has been observed in immortalized airway epithelial cell lines [20,21,22,23,43], these models often fail to accurately reflect the complex metabolic changes occurring in vivo [14,24,25]. Recent studies using primary human cells offer a more reliable indication that RSV increases glycolysis, at least in vitro [26]. However, the limited availability, donor-to-donor variability, and short-term viability of primary human respiratory cells pose significant challenges [44]. To address these limitations, we quantified the bioenergetics of live nasopharyngeal aspirate cells from infants infected with RSV, evaluated RSV-induced metabolic changes in three different relevant primary airway cell types, and utilized pediatric tonsil-derived tissue epithelium cultures as a novel in vitro model of RSV infection. Our study provides compelling evidence that RSV drives metabolic reprogramming in pediatric airways, both in situ and in vitro, supports a growing foundation for host-directed therapeutic development, and may have prognostic utility in determining whether severe disease will develop in infants [45].

The theory that viruses manipulate host cell metabolism to support their replication and survival is well established. Our previous and current observations of increased glucose uptake visualized by PET in the lungs of recently infected children provide strong evidence supporting this theory, demonstrating that enhanced glycolytic activity occurs deep within the respiratory tract in situ. Our data shows that RSV-infected patients exhibited hyperactive glycolytic foci in their lungs, with glucose uptake persisting for a remarkably extended period compared to influenza-infected patients (Figure 1e,f). This long-lasting metabolic reprogramming in situ has not been well documented previously. However, a recent study found that even after culturing cells from nasal brushings of RSV-infected infants for several days, they continued to display altered metabolic phenotypes compared to cells from uninfected children [27]. Notably, these cells were collected one year after the initial infection, suggesting that RSV can imprint lasting metabolic reprogramming in the airway epithelium. Interestingly, our PET data and the clustering of one patient 21 days after infection with the uninfected controls indicate that at the tissue level, glucose uptake and metabolites may return to homeostasis after the infection is resolved (Figure 1 and Figure 2, respectively), as observed in a mouse model where lung tissue returned to metabolic homeostasis after the second week of RSV infection [46].

Infants with acute RSV exhibited significant alterations in six metabolites detected in upper respiratory fluids compared to uninfected controls (Figure 2c). Specifically, the concentrations of citrate, malate, and D-gluconate were notably higher in RSV-infected infants. Citrate and malate are produced in the TCA cycle, and their levels in the lungs are higher than other TCA intermediates [47], while D-gluconate is involved in the pentose phosphate pathway. Similarly, in vitro infections of airway epithelial cells with respiratory viruses like IAV, SARS-CoV-2, and RSV have demonstrated increased formation of malate or citrate [26,48], suggesting enhanced flux through the TCA cycle, with one study also reporting an increase in the pentose phosphate pathway during RSV infection [26]. Conversely, the nasopharyngeal fluids of infected infants exhibited decreased levels of taurine, guanosine, and lactate. RSV and SARS-CoV-2 infections have been associated with reduced taurine levels in the urine of children and blood, respectively [16,49,50,51]. Given taurine’s cytoprotective antioxidant properties, its depletion may indicate increased oxidative stress or disrupted redox homeostasis, potentially exacerbating tissue injury or inflammation in the infected mucosa. Taurine’s antioxidant functions also help maintain mitochondrial integrity [52] and its decreased levels could impair the cell’s ability to control mitochondrial membrane integrity, contributing to the reduced respiratory capacity observed in the NPA cells (Figure 4a). The decrease in guanosine, a key purine nucleoside, could reflect high demand for nucleotide synthesis driven by viral replication. The reduction in lactate was unexpected, as previous in vitro RSV infections of A549 and small alveolar epithelial cells had found it to be increased [20,26]. The decreased lactate levels in situ may have been mediated by increased lactate clearance, or more likely, a diversion of pyruvate into the mitochondria for OXPHOS and anabolic mitochondrial pathways like glutaminolysis.

To determine the impact of RSV on intracellular metabolic reprogramming in the upper airways, we quantified and characterized the metabolism of live nasopharyngeal cells from infants with RSV infection. We previously attempted this using cryopreserved cells but found that the mitochondrial damage and metabolic instability from freeze–thaw overwhelmed the infection-induced effects. To avoid this, we immediately analyzed the metabolism of fresh nasal aspirates from infected infants, allowing us to demonstrate that RSV strongly and significantly increases glycolysis and respiration in the upper airway cells in situ (Figure 3a and Figure 4a). Fresh nasal aspirates contain a complex mixture of epithelial and immune cells [53,54], with each cell type exhibiting distinct metabolic responses to infection, posing a challenge in attributing the changes to specific pathways or cell types. Consistent with these findings, our data indicates that these samples are complex, predominantly composed of immune cells such as monocytes, dendritic cells, T cells and B cells, and epithelial cells. To delineate the epithelial contribution, we utilized two in vitro models: human tonsil epithelium cultures and human bronchial epithelial cells from pediatric donors. Human tonsil epithelium cultures are a new infection model [29] that is highly relevant in the context of respiratory infections including RSV [55,56,57,58]. These approaches enabled us to evaluate RSV-induced metabolic reprogramming in the nasal cavity and pharynx using the patient-derived upper respiratory cells, as well as in the upper and lower airway epithelial cells using the in vitro models.

Infant upper respiratory cells infected in situ, as well as pediatric tonsil epithelium cultures and human bronchial epithelial cells infected with RSV in vitro, all demonstrated a significant escalation in glycolysis compared to controls (Figure 3). These findings align with previous studies showing increased glycolysis in RSV-infected airway epithelial cells [20,21,22,23,26,27]. The infected cells exhibited a surge in glycolytic capacity, suggesting a greater ability to upregulate glycolysis when energy demands are high, or mitochondrial ATP production is compromised [59]. Glycolytic reserve was also significantly increased in URCs from RSV patients and RSV-infected TEpiCs, indicating these infected upper airway cells were not only using glycolysis more under basal conditions but were also primed to further increase glycolysis under stress, a sign of increased metabolic plasticity and reprogramming driven by RSV. In contrast, lower airway epithelial cells displayed increased glycolysis in response to RSV but did not show the same increase in glycolytic reserve, potentially reflecting reduced metabolic flexibility or a more constrained reprogramming response. Overall, these findings support a consistent RSV-driven induction of glycolytic metabolism across infant samples and in vitro pediatric airway models, which may influence local immune responses, viral replication dynamics, and disease severity.

In addition to enhanced glycolysis, RSV-infected upper respiratory cells from infants showed significantly elevated basal mitochondrial respiration, that did not increase ATP production, and reduced spare respiratory capacity compared to uninfected controls (Figure 4a), suggesting a metabolically strained yet highly active state. The increase in basal respiration aligns with prior in vivo data [46,60], and suggests heightened mitochondrial activity potentially reflecting increased biosynthetic demand during infection. Importantly, the marked reduction in spare respiratory capacity, a sensitive indicator of mitochondrial fitness or dysfunction [25,61,62], implies that the infected URCs are operating near their respiratory limit and may have diminished capacity to respond to additional stress. In contrast, RSV-infected pediatric tonsil epithelial cells displayed increased maximal respiration and spare capacity without changes in basal respiration, suggesting a more resilient mitochondrial phenotype. These differences may reflect the presence of immune cells within the URC population or unique metabolic rewiring of the upper respiratory epithelium by RSV. Collectively, these findings point to RSV-driven metabolic reprogramming in pediatric airway cells, characterized by heightened glycolysis and strained mitochondrial function, which may impact viral replication, host defense, and disease severity.

This study represents the first quantification of the bioenergetics of nasopharyngeal cells from patients infected with RSV or other respiratory viruses, the initial evaluation of RSV-induced metabolic changes in three distinct primary airway cell types, and the pioneering use of nasopharyngeal tonsil tissue epithelium cultures as an in vitro model of RSV infection. In light of these initial findings, it is now possible to argue that RSV induces global changes in tissue metabolism across the lungs and upper airways, and reconfigures intracellular metabolism in situ, which opens new avenues for targeted therapeutic development. Our previous research found that infants with severe RSV infection exhibited distinctively elevated levels of the cytokines IL-1β and IL-33, which were observed to modify lung epithelial stem and progenitor cells in a neonatal mouse model [63,64]. These cytokines are known to increase glucose uptake and glycolysis in epithelial and immune cells [65,66,67,68,69,70,71], potentially amplifying viral replication and aggravating inflammation within infant airways. A limitation of the current study is the reduced sample size due to patient specimens not meeting quality control standards, which may constrain the generalizability of the findings and potentially limit statistical power. Further investigations with larger sample sizes are necessary to corroborate the results, explore the impact of metabolic reprogramming on viral load, illness severity/duration, and long-term protection, and determine the influence of IL-1β and IL-33 on RSV-induced metabolic changes in infants.

## 5. Conclusions

Our findings provide compelling evidence that respiratory syncytial virus induces metabolic reprogramming in pediatric airway cells, both in situ and in vitro. We observed marked glycolytic activation in the lungs of children infected with RSV, as well as in upper airway cells from infants and in pediatric airway epithelial cultures infected with the virus. These results align with and expand upon prior studies, demonstrating that RSV promotes a metabolic shift toward glycolysis across multiple compartments of the respiratory tract. Importantly, this work introduces two key methodological advances: the capability to quantify live-cell metabolism in freshly collected infant nasopharyngeal aspirates, and the establishment of pediatric-tonsil-derived epithelial cell cultures as a novel, physiologically relevant in vitro model for studying RSV infection. Together, these tools and findings offer new insights into the complex virus–host interactions occurring at tissue, cellular, and metabolic levels. By identifying and characterizing virus-driven changes in host metabolism, this research supports the development of host-directed therapeutic strategies. Given that metabolic reprogramming is a hallmark of many viral infections, the pathways uncovered here represent promising targets for broadly effective antiviral interventions.

## Data Availability

The raw data supporting the conclusions of this article will be made available by the authors on request.

## Supplementary Materials

The following supporting information can be downloaded at: https://www.mdpi.com/article/10.3390/v17050703/s1, Figure S1: Characterization of TEpiCs by flow cytometry, Figure S2: Statistical summary of metabolic responses to RSV infection in primary airway cells using mixed-effects modeling.

## Abbreviations

ATP	Adenosine Triphosphate
ECAR	Extracellular Acidification Rate
FDG	Fluorodeoxyglucose
HBECs	Human Bronchial Epithelial Cells
IL-1β	Interleukin-1 Beta
IL-33	Interleukin-33
NPA	Nasopharyngeal Aspirates
OCR	Oxygen Consumption Rate
OXPHOS	Oxidative Phosphorylation
PET	Positron Emission Tomography
RSV	Respiratory Syncytial Virus
RT-qPCR	Reverse Transcription Quantitative Polymerase Chain Reaction
TEpiCs	Tissue-Derived Multicellular Epithelial Cultures
TCA	Tricarboxylic Acid
URCs	Upper Respiratory Cells
