# Supplementary material for: Respiratory Syncytial Virus Elicits Glycolytic Metabolism in Pediatric Upper and Lower Airways
Source: Viruses. 2025 May 14;17(5):703. doi: 10.3390/v17050703 (PMC12115633; doi:10.3390/v17050703)
Supplement: Supplementary file 1 [file viruses-17-00703-s001.zip › viruses-3521027-supplementary.pdf]

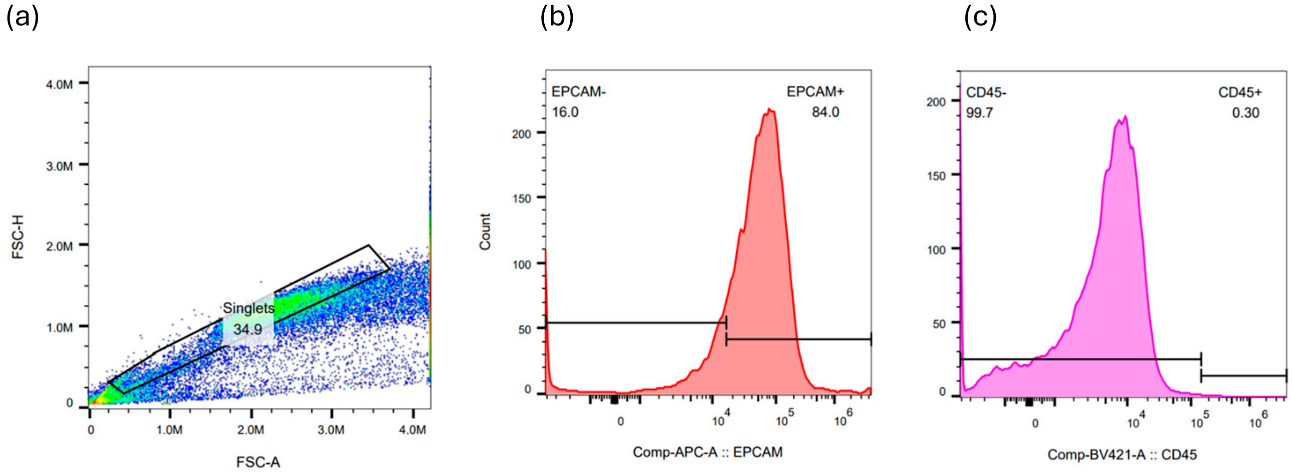

Supplementary Figure S1. Characterization of TEpiCs by flow cytometry. Tonsils were obtained from pediatric patients as a part of standard care procedures. Cells were processed, enumerated and cryopreserved. Thawed cells were cultured in BEGM until they reached 80% confluency. Cells were stained with antibodies against EpCAM and CD45 on the Aurora spectral flow cytometer and the data were analyzed with FlowJo software. (a) Single-cell selection. (b) Histogram showing EpCAM expression, confirming the epithelial identity of the cultured cells. (c) Histogram showing CD45 expression, indicating the absence of immune cell contamination. Data shown are representative of three independent biological replicates.

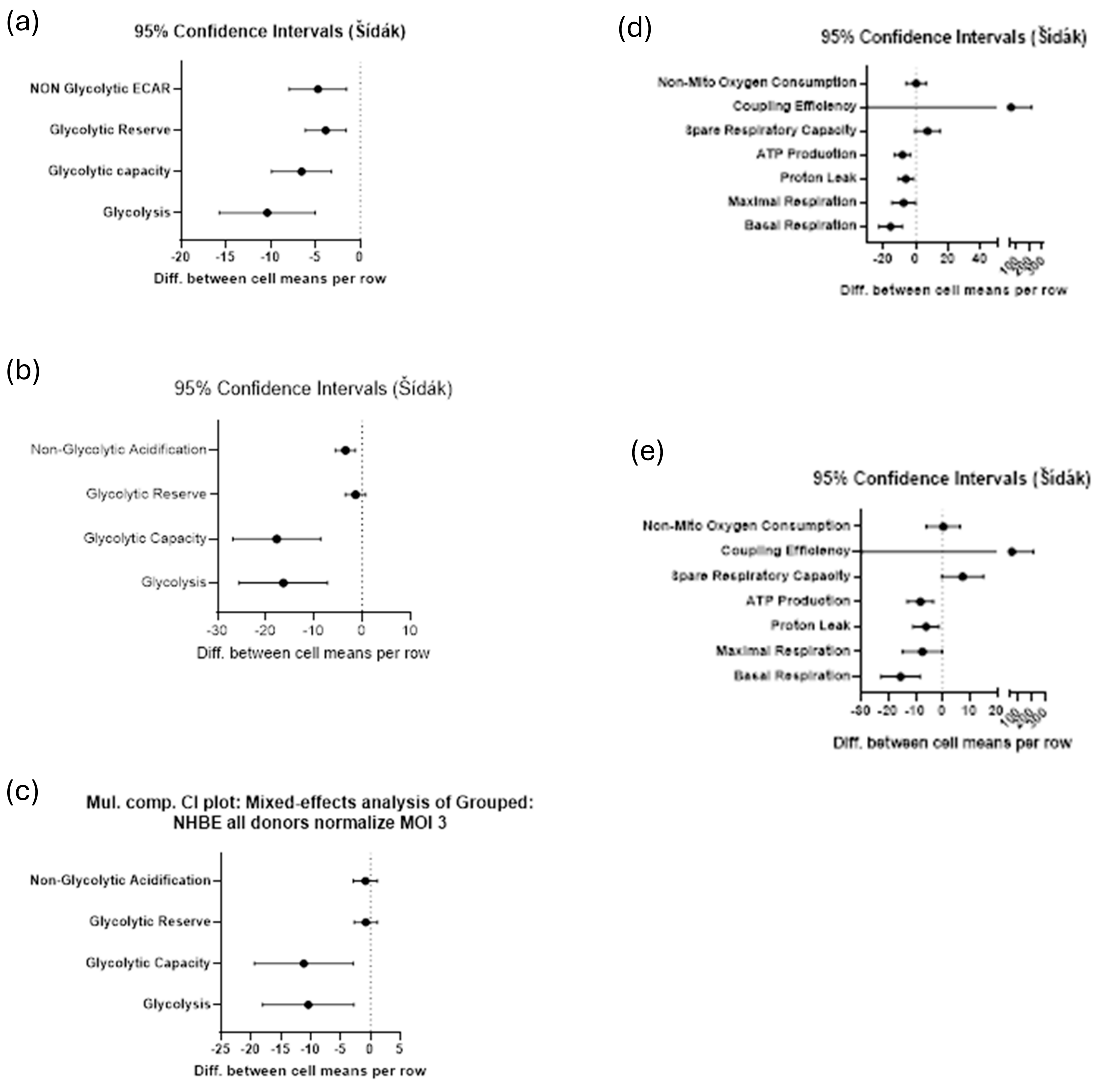

Supplementary Figure S2. Statistical summary of metabolic responses to RSV infection in primary airway cells using mixed-effects modeling. 95% confidence intervals (Sidak-corrected) showing the difference in means between RSV-infected and control conditions for each metabolic parameter. (a) ECAR-derived glycolytic parameters in URCs. (b) ECAR-derived glycolytic parameters in TEpiCs. (c) ECAR-derived glycolytic parameters in HBECs (d) OCR-derived mitochondrial parameters in URCs. (e) OCR-derived mitochondrial parameters in TEpiCs. Parameters were assessed using a two-way repeated measures mixed-effects model with post hoc Sidak correction for multiple comparisons.
